# Outcomes research in non-specific low back pain: Knowledge transfer in clinical practice

**DOI:** 10.1007/s00508-019-1523-4

**Published:** 2019-06-24

**Authors:** Tanja A. Stamm, Anna Boesendorfer, Maisa Omara, Valentin Ritschl, Siniša Štefanac, Erika Mosor

**Affiliations:** 1 Section for Outcomes Research, Center for Medical Statistics, Informatics, and Intelligent Systems, Medical University of Vienna, Spitalgasse 23, 1090 Vienna, Austria; 2 Ludwig Boltzmann Institute for Arthritis and Rehabilitation, Nußdorfer Straße 64, 1090 Vienna, Austria

**Keywords:** Patient-reported outcomes, Outcome measures, Adherence, Non-pharmacological treatment, New technologies

## Abstract

**Objective:**

The aims of this article are to provide an overview and discuss current concepts and future trends in outcomes research in non-specific low back pain, specifically considering the perspective of patients, patient-reported outcomes and outcome measures as well as to facilitate knowledge transfer into clinical practice.

**Review strategy:**

The breadth of this work and the required brevity of this article were not amenable to a formal approach, such as a systematic literature review or a formal scoping review. Literature sources were identified through medical databases but different sources of information and of various methodologies were also included. Furthermore, outcomes meaningful for patients and examples of outcome measures that are applicable in clinical practice were extracted. Areas for future research were identified and discussed.

**Results:**

Patient-reported outcomes and outcome measures are essential in patient-centered care. The assessment of the patients’ perspective is important to ensure motivation, active involvement, self-management and adherence, especially in non-pharmacological interventions for low back pain. To facilitate the use of outcome measurements for low back pain in clinical practice, future studies should focus on a clinically feasible index, which includes patient-reported as well as clinician-reported or performance-based variables. Relationships between different types of outcomes and outcome measures as well as resource and outcome-based healthcare constitute important topics for future research. New digital technologies can support continuous outcome measurement and might enable new patient-driven models of care.

**Conclusion:**

Active patient involvement is an essential part of non-pharmacological treatment in low back pain and needs to be considered in terms of outcomes and outcome measurement.

## Introduction

Due to the increased longevity of the population in industrialized countries, global priorities in healthcare have been shifting from communicable to non-communicable diseases [1]. Musculoskeletal conditions are the main burden of non-communicable disease-related disability [2]. Reports on global burden of diseases have extended the scope of musculoskeletal conditions and identified low back pain as one of the highest burdens of disability in all specific conditions assessed [3, 4]. Low back pain is often recurrent and non-specific and considered to be a multifactorial condition. The most common form of low back pain is non-specific low back pain. This term is used when the pathoanatomical cause of the pain cannot be determined [5, 6].

The lifetime prevalence of low back pain is estimated at 60–70% in industrialized countries. Low back pain affects the population ranging from children to older adults, furthermore the prevalence rate for children and adolescents could reach that which is seen in adults [7]. In 2010 age-standardized prevalence was found to be highest in western Europe [4]. Thus, low back pain is considered to be a serious public health concern. In 2016, low back pain was estimated to be one of the leading causes of disability-adjusted life years (DALYs) in most countries and territories [2]. The DALY is the standard metric used to quantify the burden of a disease and is calculated by combining years of life lost in a population due to premature mortality and years lived with disability. One DALY represents the loss of the equivalent of 1 year of full health [8]. During a 6-month period, 72% of adults in the general population reported low back pain and of these, 11% had disabling low back pain [3, 9]. Low back pain is particularly frequent in people at the age of 35–55 years. Thus, low back pain has a severe impact on the workforce and constitutes a considerable socioeconomic impact in terms of working days lost [10]. Low back pain caused by other reasons, such as malignancies or disc prolapse were excluded from these analyses as well as from this review, which focuses on non-specific low back pain.

Outcomes in health care comprise the measurement of clinical signs and symptoms as well as the results of medical interventions but also include quality of life, functioning, pain, fatigue or the impact of exercise-induced dyspnea in daily life, outcomes which are often most important for patients. In people with acute and chronic health conditions, in children, in older adults and in rehabilitation, it is essential to include the perspective of patients into outcome measurements. Outcomes research constitutes a relatively young scientific discipline with a broad focus; however, it applies some common underlying principles, such as including the patient perspective, patient-reported outcomes and outcome measures to capture what matters to patients [11]. Considering the perspective of patients is essential especially in diseases, such as low back pain, which impact on different aspects of human life and treatment commonly requires behavioral change driven by the motivation of the patients. Active patient involvement not only in the management but also in terms of outcome measurement, is thus an essential requirement. Therefore, the aim of this work was to (i) provide an overview and discuss current concepts and future trends in outcomes research in low back pain, specifically considering the perspective of patients and patient-reported outcomes and outcome measures, as well as facilitate knowledge transfer into clinical practice.

## Review strategy

The breadth of this work and the required brevity of this article were not amenable to a formal approach, such as a systematic literature review or a formal scoping review. Different sources of information and various methodologies ranging from scientific articles to policy documents and expert opinions were included. Documents were obtained from the National Institutes of Health National Library of Medicine (PubMed) using the following keywords and their combinations: patient-reported outcome, functioning, low back pain, patient perspectives, (non-pharmacological) interventions. Furthermore, policy documents and reports on the global burden of illness were reviewed, as well as textbooks regarding outcomes and outcome measures. The titles and abstracts of journal articles and summaries of other documents identified from this search were assessed for eligibility, alongside grey literature cited in these articles. Grey literature refers to literature published outside of the traditional academic dissemination channels and includes project/evaluation reports, working papers, policy documents, opinion papers, etc. Outcomes meaningful for patients, examples of outcome measures that are applicable in clinical practice and interventions for low back pain were extracted. Potential questions for future research were identified and discussed. Due to the variety of literature included and the exploratory nature of this review, no formal inclusion or exclusion criteria for the literature were defined.

To select guidelines, the authors conducted a literature search in PubMed, Cumulative Index to Nursing and Allied Health Literature (CINAHL) and the Cochrane Library to search for clinical guidelines for low back pain from the last 5 years using the following keywords (in title) “guideline” AND “low back pain” or “LBP”. Reviews and studies that gave an overview on guidelines on low back pain were screened for the original guidelines and possible updates of these. The following inclusion criteria were defined: a) coverage of non-pharmacological methods in the management of low back pain, b) latest version of the guidelines not older than 5 years (as recommended by the National Institute for Health and Care Excellence [12]) and c) English and/or German version available. The following exclusion criteria were defined: a) guidelines based on already included guidelines or a synopsis of already included guidelines, b) no clear description of the development process and c) guidelines focusing on a specific area, such as chiropractic, or pharmacological treatment only.

## Patient-reported outcomes and outcome measures

Low back pain is associated with several important outcomes, such as functionality, pain and health-related quality of life. Patient-reported outcome measures are commonly accepted to evaluate the outcome of low back pain interventions; however, there is still a lack of consensus on what measures to use for assessing outcomes that are important for patients with low back pain and other stakeholders, such as health professionals and employers, and which outcome measure(s) best capture the effectiveness of pharmacological and non-pharmacological interventions [13].

In a disease where by definition no anatomically correlates can be objectified and which affects up to 72% of the adult population at least once, self-reported outcome measures should be used in combination with clinician-reported outcome measures, e.g. in form of a combined index, rather than relying solely on self-reported instruments to assess health outcomes of patients.

The patterns of onset of back pain varies within the different spinal regions, age, as well as between genders [7]. Accordingly, it is important to differentiate outcomes among genders, spinal regions and age groups, to further understand the onset, nature and course of the disease and to better customize prevention and treatment strategies. Furthermore, low back pain is characterized by acute and chronic pain, with and without leg pain and a range of pain severity including mild, moderate, and severe pain. Previously, the diagnosis of low back pain was reported separately independent from its functional impact; however, the establishment of the International Classification of Functioning, Disability and Health (ICF) made it possible to explicitly link disease coding with outcomes of functioning in daily life [3, 14]. In 2002, the comprehensive and brief ICF core sets for patients with low back pain were created, which include the most typical ICF categories that are affected in low back pain and define what should be measured to comprehensively represent the experience of patients. The selected categories can be taken into account when conducting a comprehensive, multidisciplinary assessment [15]. Moreover, the International Consortium for Health Outcomes Measurement (ICHOM) provides a standard set for measuring outcomes of low back pain patients [16, 17]. New e‑health technologies, such as apps or innovative information and communication technologies can support continuous outcome measurement between clinical visits and might enable new, patient-driven models of care [18]; however, there is so far no simple clinically feasible index combined from patient-reported and clinician-reported variables.

Table 1 provides examples for frequently used patient-reported outcomes and outcome measures in low back pain which could be implemented into clinical practice [13, 16, 19–21].

**Table 1:** Examples for patient-reported outcome measures that assess chronic low back pain symptoms [13, 16, 19–21]

Instrument	Outcome	Description
Oswestry Disability Index version 2.1a (ODI 2.1a)	Physical functioning	The ODI is a recommended condition-specific patient-reported outcome measure used to evaluate functional status in patients with back pain. It consists of 10 items: pain intensity, personal care, lifting, walking, sitting, standing, sleeping, sex life, social life and travelling.
24-item Roland Morris Disability Questionnaire (RMDQ-24)	Physical functioning	The RMDQ consists of 24 statements about activity limitations due to back pain, e.g. self-care and walking.
Visual Analogue Scale (VAS) of back pain intensity and Pain Numerical Rating Scale (PNRS)	Pain	A VAS and PNRS ask the patient to rate pain from 0 to 10 (11 point scale) or from 0 to 100 (101 point scale) where 0 is equal to no pain and 10 or 100 is equal to worst possible pain.
Short Form Health Survey 12 (SF-12)	Health-related quality of life	The SF-12 Health Survey is a 12-item questionnaire used to assess generic health outcomes from the patient’s perspective.
Work Ability Index (WAI)	Work ability	The WAI is a validated instrument that assesses the individual work ability. Through a self-assessment questionnaire, the WAI measures a worker’s work ability and helps to define necessary measures for maintaining and promoting work ability, as it enables detecting work-related health risks as early as possible.
Work Limitations Questionnaire (WLQ)	Work ability	The WLQ assesses the on-the-job impact of chronic health problems and/or treatment (work limitations).
Canadian Occupational Performance Measure (COPM)	Activities of daily life, participation	The COPM is an individualized, client-centered outcome measure; designed to capture a patient’s self-perception of performance in everyday activities over time.

## Patient-reported outcomes in clinical trials

Several updates on how to establish core set of outcome domains and measures for low back pain in clinical trials [22–26] resulted in a consensus of three main areas: physical functioning, pain intensity and health-related quality of life [27]. The latest recommendation that resulted from a systematic review [28] is to use the Oswestry Disability Index version 2.1a or the 24-item Roland Morris Disability Questionnaire for physical functioning, an 11-point numeric rating scale referring to average low back pain intensity over the last week for pain intensity, the Short Form Health Survey 12 or the 10-item Patient-Reported Outcomes Measurement Information System Global Health form for health-related quality of life [19]; however, many authors [29] suggest adding further domains to the assessment of low back pain, such as measuring current work ability and psychological functioning.

## Patient-reported outcomes versus performance-based measurements of functioning

A patient-centered personalized care in low back pain calls for the use of patient-reported outcomes and outcome measures to assess the impact of the disease in daily life; however, more objective, functional, performance-based measurements complement the use of patient-reported data. A broad range of functional assessments exists, e.g. functional assessments in the area of occupational health and paid work. This review focused on patient-reported outcomes and outcome measures. Future research should look more closely into the relationship between patient-reported and performance-based instruments and examine which areas of functioning each instrument covers, and whether patient-reported outcome predicts performance-based measurements and vice versa. Clinical tests for low back pain are often used to a great extent, while various patient-reported outcome measures are seldom used in clinical practice [30]. The motivation of patients plays an important role also in the assessment but performance-based instruments often do not cover the range of problems that patients experience in daily life. Instead, they often focus on specific aspects of functioning [31, 32], e.g. motor coordination or the ability to change basic body positions.

## Outcomes and outcome measures related to non-pharmacological treatment in low back pain

Patient-reported outcomes and outcome measures are essential in non-pharmacological interventions as the motivation, active involvement and self-management skills of patients are crucial. Improvement in general health outcomes and positive experiences of patients might also be related to the use of patient-reported outcome measures and could also serve as a communication tool between patients and clinicians [33, 34]. Therefore, measuring health outcomes in people with low back pain plays an important role for developing and utilizing more evidence-based healthcare decisions on prevention and treatment [35]. Recent management guidelines from Denmark, Germany, United Kingdom (UK) and two from the United States of America (USA), guidelines from the American College of Physicians (ACP) and another from the Institute for Clinical Systems Improvement (ICSI), highlight the importance of non-pharmacological treatment as part of the management of low back pain [36–40]. Non-pharmacological treatment is recommended as an initial treatment for acute, subacute and chronic low back pain. According to the guidelines, pharmacological treatment should only be selected if patients desire medication and/or chronic low back pain has an inadequate response to non-pharmacological therapy [38]. The content of these guidelines is comparable with a few exceptions, e.g. the benefits from the use of acupuncture are controversial (Tables 2 and 3); however, the comparison of various guidelines for the management of low back pain showed that patient-reported outcome measures are only briefly mentioned.

**Table 2:** Overview of the level of evidence of non-pharmacological methods in the management of low back pain. If the levels of evidence were explicitly reported in the guidelines they were extracted and are mentioned in this table

Intervention	Guidelines	Level of evidence
*Self-management*		
Advice to stay active: maintaining usual levels of daily activity despite pain, including work [37]	Denmark [37]^a,b^	Low
	Germany [40]	Good to weak
	UK [36]	Moderate to very low
	USA_ACP [38]^a,b^	Not reported
	USA_ICSI [39]^a,b^	Moderate
Patient education: regarding health literacy, competencies, and adaptation of behavior [37]	Denmark [37]^a,b^	Very low
	Germany [40]	Good to weak
	UK [36]	Moderate to very low
	USA_ACP [38]^a,b^	Not reported
	USA_ICSI [39]^a,b^	High to moderate
*Manual therapy*		
Spinal manual therapy: any mobilization or spinal manipulation technique [37]	Denmark [37]^a,b^ (in addition to usual care)	Low
	USA_ACP [38]	Low
	USA_ICSI [39]^a,b^	Moderate to low
Manual therapy (spinal manipulation, mobilization or soft tissue techniques such as massage) in combination with exercise, with or without psychological therapy	UK [36]	High to very low
Massage	USA_ACP [38]^a,b^	Low
*Exercise*		
(Supervised) exercise: individualized exercises or physical activity (e.g. back-specific strengthening, stretching, motor control exercise or mobilizing exercises and cardiovascular training) delivered by a trained healthcare professional [37]	Denmark [37]^a,b^	Low
	USA_ACP [38]^c^	Moderate, motor control therapy: low
Movement therapy, including educative approach	Germany [40]^b,c^	Good to weak
Group exercise	UK [36]	Moderate to very low
	USA_ACP [38]^c^	Moderate
Sport rehabilitation program or functional training (if the limitation in daily activities remains and the occupational rehabilitation is at risk)	Germany [40]^b,c^	Expert consensus
Tai chi	USA_ACP [38]^c^	Low
Yoga	USA_ACP [38]^c^	Low
*Psychological therapy and/or interventions*		
Psychological therapy (using a cognitive behavioral approach) in combination with other treatments (e.g. exercise, manual therapy) or a multimodal program	Germany [40]^c^	Good to weak
	UK [36]	Moderate to very low
Cognitive behavioral therapy	Germany [40]^b^ (if psycho-social risk factors exist)	Weak
	USA_ACP [38]^c^	Low
Operant therapy	USA_ACP [38]^c^	Low
Mindfulness-based stress reduction	USA_ACP [38]^c^	Moderate
Progressive relaxation	Germany [40]^c^	Weak
	USA_ACP [38]^c^	Low
*Multidisciplinary programs*		
Multidisciplinary rehabilitation: combines a physical and psychological program	UK [36] (if significant psychosocial obstacles to recovery exist or previous treatments have not been effective)	Moderate to very low
	USA_ACP [38]^c^	Moderate
*Return to work*		
Return to work programs (work or normal activities of daily living)	UK [36]	High to very low
*Acupuncture*		
Acupuncture	USA_ACP [38]	Low^a, b^, moderate^c^
	USA_ICSI [39]^a,b^	Low
*Biofeedback, laser therapy*		
Electromyography biofeedback	USA_ACP [38]^c^	Low
Low-level laser therapy	USA_ACP [38]^c^	Low
*Others*		
Superficial heat	USA_ACP [38]^a,b^	Moderate
	USA_ICSI [39]^a,b^	Moderate
Cryotherapy	USA_ICSI [39]^a,b^	Expert consensus

The guidelines used different modified versions of the Grading of Recommendations Assessment, Development and Evaluation (GRADE): Denmark (high, moderate, low, very low) [37], Germany (weak that refers to very low-low, good that refers to moderate-high) [40, 41], UK (high, moderate, low) [36], USA_ACP (high, moderate, low) [38], USA_ICSI (high, moderate, low, very low) [39]

*USA_ACP* American College of Physicians, *USA_ICSI* Institute for Clinical Systems Improvement

^a^For acute low back pain patients

^b^For subacute low back pain patients

^c^For chronic low back pain patients

**Table 3:** Overview of non-recommended non-pharmacological methods in the management of low back pain

Intervention	Guidelines
*Manual therapy*	
Massage	Germany [40]*
Traction	UK [36]
Motorized traction	Germany [40]
*Work hardening*	
Work hardening/work conditioning	Germany [40]*
*Acupuncture*	
Acupuncture	Denmark [37]
	UK [36]
*Ultrasound, electrotherapies, laser therapy, magnet therapy, diathermy*	
Ultrasound	Germany [40]
	UK [36]
Transcutaneous electrical nerve stimulation (TENS)	Germany [40]*^,+^
	UK [36]
Percutaneous electrical nerve stimulation (PENS)	Germany [40]
	UK [36]
Interferential therapy	Germany [40]
	UK [36]
Laser therapy	Germany [40]
Magnet therapy	Germany [40]
Short-wave diathermy	Germany [40]
*Others*	
Cryotherapy	Germany [40]
Orthotics (such as belts, corsets, foot orthotics, rocker sole shoes)	Germany [40], specifically (foot) orthotics
	UK [36]
Kinesio taping	Germany [40]

*USA_ACP* American College of Physicians,* USA_ICSI* Institute for Clinical Systems Improvement

*For acute low back pain patients

^+^For chronic low back pain patients

Self-management techniques highlight the importance of a patient-centered approach. Self-management includes information on, e.g. causes of low back pain, patient education, the advice to stay active despite pain [36–40], and to avoid bed rest [40]. Of the guidelines four recommend manual therapy, such as manipulation, mobilization or soft tissue techniques [36–39] to reduce pain and improve function. Manual techniques are defined by the UK guidelines as “spinal manipulation (a gapping motion of a synovial joint within a spinal segment in response to a force of typically short duration), spinal mobilisation (joint movement within the normal range of movement) and soft tissue techniques (manual manipulation/mobilisation of soft tissues)” [36]. Spinal manual therapy is defined by the Danish guidelines as “any manual technique that moves one or more joints within normal ranges of motion and aims at improving spinal joint motion or function, i.e., any mobilisation or spinal manipulation technique” [37]; however, the benefits of manual therapy as a single intervention are not yet clear. Therefore, manual therapy should always be offered in combination with other interventions, such as exercises with or without psychological interventions [36] and/or in addition to usual care [37]. Traction [36] and motorized traction [40] are not recommended as interventions for low back pain patients. There are controversial statements about massage for acute and subacute low back pain patients. The German guidelines do not recommend massage for acute low back pain patients [40], whereas one of the US guidelines [38] does recommend massage for acute and subacute low back pain patients. For the management of low back pain supervised [37], individually designed exercises (including, but not limited to tai chi, yoga, movement therapy including educative approach and motor control [37, 38, 40]) and group exercises [36, 38] are recommended to reduce pain and to improve function. Additionally, if the limitation in daily activities remains and the occupational rehabilitation is at risk for subacute and chronic low back pain patients, sport rehabilitation programs or functional training are recommended [40]. Likewise, mindfulness-based stress reduction, cognitive behavioral therapy, operant therapy [38] and progressive relaxation [38, 40] should be considered for patients with chronic low back pain as single strategies to reduce pain and improve function [38], and also for patients with subacute low back pain, if psychosocial risk factors exist [40]. Other psychological interventions, such as cognitive behavioral approaches can be considered for some patients in combination with other treatments (e.g. exercise, manual therapy [36]) or for chronic low back pain patients within a multimodal program (multimodal pain management and rehabilitation) [40]. In cases of psychosocial obstacles, non-beneficial singular interventions [36] and/or patients suffering from a chronic type of back pain [38], a multidisciplinary rehabilitation program/intervention is recommended. Counselling and/or formal programs that support a return to work and/or other activities of daily living were found to be important for the management of low back pain [36]. Acupuncture is controversially discussed in the literature. Whereas the ACP [38] and the ICSI [39] recommend acupuncture to be used for acute, subacute [38, 39] and chronic low back pain [38], Stochkendahl et al. [37] and the National Institute for Health and Care Excellence [36] advised against it. Superficial heat as an intervention for patients with acute or subacute low back pain is suggested in the American guidelines [38, 39] only. Cryotherapy is controversial when comparing the guidelines, it is recommended for acute and subacute low back pain by the ICSI [39], whereas the German guidelines [40] recommend against cryotherapy. Electromyography biofeedback is also recommended for patients with chronic low back pain [38], whereas ultrasound, percutaneous electrical nerve stimulation (PENS), transcutaneous electrical nerve stimulation (TENS), interferential therapy [36, 40], magnet therapy and short-wave diathermy [40] should not be part of treatment in low back pain. The recommendations on laser therapy are controversial. The ACP [38] recommends low-level laser therapy for patients with chronic low back pain, whereas the German guidelines [40] recommend against laser therapy in the management for low back pain. Likewise, various types of orthotics, such as (foot) orthotics [36, 40], belts, corsets, or rocker sole shoes [36], and kinesio taping [40] are not recommended for the management of low back pain. For patients with acute low back pain, the German guidelines do not recommend work hardening and work conditioning [40]. Taken together, active patient involvement is an essential part of non-pharmacological treatment in low back pain and needs to be considered in terms of outcomes and outcome measurement.

## Conclusion

Patient-reported outcomes and outcome measures are essential in patient-centered care. Especially in non-pharmacological interventions for low back pain, assessment of the perspective of patients is important to ensure motivation, active involvement, self-management and adherence. To facilitate the use of outcome measurements for low back pain in clinical practice, future studies should focus on a clinically feasible index which includes patient-reported, along with clinician-reported or performance-based variables. Relationships between different types of outcomes and outcome measures as well as resource and outcome-based healthcare constitute important topics for future research. New digital technologies can support continuous outcome measurement between clinical visits and might enable new, patient-driven models of care.

## Appendix

### Description how the levels of evidence were determined

The levels of evidence in the manuscript were extracted from the guidelines, if they were explicitly mentioned. The guidelines used modified versions of the Grading of Recommendations Assessment, Development and Evaluation (GRADE) [42], but with different definitions and numbers of categories. The Danish guidelines [37] assessed the quality of evidence according to an adapted version of GRADE [42] (high, moderate, low, very low). The levels of evidence of the German guidelines [40, 41] are based on the procedure of GRADE [42] and assessed according to the Oxford Centre for Evidence-Based Medicine [43]. The data quality, which is reported in Table 2, is reported as weak (very low-low) or good (moderate-high). The UK guidelines [36] also use a modified version of the GRADE toolbox [44] to assess the evidence for outcomes of intervention reviews (high, moderate, low). The US guidelines from the ACP [38] uses the ACP guideline grading system [45], which is adopted from the GRADE procedure [46] to assess the quality of evidence (high, moderate, low). The US guidelines from the ICSI [39] uses also a modified version of the GRADE methodology system [44] (level of evidence: high, moderate, low, very low).
